# Urbanization-induced soil organic carbon loss and microbial-enzymatic drivers: insights from aggregate size classes in Nanchang city, China

**DOI:** 10.3389/fmicb.2024.1367725

**Published:** 2024-02-28

**Authors:** Foyi Zhang, Jialin Zhong, Yuquan Zhao, Changyongming Cai, Wei Liu, Qiong Wang, Wenjie Wang, Huimei Wang, Xueru Jiang, Renqiang Yuan

**Affiliations:** ^1^College of Art and Landscape/College of Forestry, Jiangxi Agricultural University, Nanchang, Jiangxi, China; ^2^State Key Laboratory of Subtropical Silviculture, Zhejiang A&F University, Hangzhou, Zhejiang, China; ^3^School of Landscape Architecture, Beijing Forestry University, Beijing, China

**Keywords:** urbanization, aggregate size, soil organic carbon, soil microbial community, soil enzymatic activity

## Abstract

Soil microorganisms and enzymes play crucial roles in soil organic carbon (SOC) sequestration by promoting soil aggregate formation and stability and by participating in SOC cycling and accumulation. However, the effects by which soil microorganisms and enzymes act as mediators driving dynamic changes in SOC during rapid urbanization remain unclear. Therefore, this study selected the built-up area of Nanchang City, China (505 km^2^), as the study area. Sampling surveys were conducted using 184 sample plots stratified based on the proportion of impermeable surface area to distinguish different urbanization levels. The driving factors of dynamic changes in SOC of different aggregates during the process of urbanization were analyzed using the soil microbial community and enzyme activities. The results demonstrated that with an increase in urbanization intensity, both SOC content and stock exhibited a significant decline (*p* < 0.05). The highest SOC stock and contribution rate were observed in the 0.25–1 mm aggregates, and they were significantly influenced by urbanization (*p* < 0.05). In addition, the biomass of gram-positive bacteria (G+) and actinomycetota, and the activities of N-acetylglucosaminidase and acid phosphatase (AP) were significantly higher in low-urbanization areas than in high-urbanization areas (*p* < 0.05). SOC of each aggregate was positively correlated with fungi, arbuscular mycorrhizal fungi, G+, gram-negative bacteria, actinomycetota, protozoa, β-1,4-glucosidase, N-acetylglucosaminidase, AP, urease, and catalase. Compared to soil enzymes, soil microorganisms exhibited a greater role in SOC sequestration (22.7%). Additionally, a structural equation model indicated that urbanization can directly or indirectly lead to a decrease in SOC of aggregates by altering soil physicochemical properties and affecting microbial and enzyme dynamics. However, the larger vegetation characteristics index mitigate the negative impacts of urbanization on SOC. Overall, urbanization had a negative impact on soil carbon storage. In the future, it is important to consider strategies that focus on improving soil nutrients, maintaining soil structure, protecting existing urban trees, and enhancing plant diversity during the urbanization process. These measures can help increase soil microbial biomass and enzyme activity, thereby improving soil and aggregate-related SOC content. The study could contribute to enhancing carbon sequestration in urban greenspaces.

## 1 Introduction

Over the past few decades, natural and rural land worldwide has been rapidly converted to urban land, and it is projected that by 2030, the urban land area will double (Seto et al., 2012). Rapid urbanization is causing global environmental changes and is expected to have severe consequences on biodiversity and natural ecological processes (Pouyat et al., 2007). Particularly in developing countries, the high demand for construction land exacerbates this process. As the world’s largest developing country, China’s urban population has surpassed 900 million, with an urbanization rate as high as 63.89% (National Bureau of Statistics of China, 2024). The rapid and intensive expansion of cities has altered land use patterns, resulting in the coverage or fragmentation of previously natural land into fragmented patches. This significantly affects greenspace soil properties and functions (e.g., land pollution, organic matter loss, biodiversity loss, and soil structural degradation), restricts vegetation growth, and poses a threat to human health (Zhang et al., 2020). Soil serves as a medium for urban green vegetation, providing essential life elements such as water, nutrients, and minerals necessary for plant growth. Its quality directly affects vegetation growth as well as the performance of landscape functions and ecological benefits. Amid the increasingly rapid pace of urbanization, it is important to protect or improve urban soil quality for healthy and sustainable urban greenspace development. Soil organic carbon (SOC) plays an important role in the soil ecosystem of urban greenspaces. They serve as a vital soil nutrient reservoir that influences plant growth and ecosystem stability in urban greenspaces. In addition, it is an integral global carbon cycle component and regulates atmospheric CO_2_ concentrations and global climate change (Zhang et al., 2021). Luo et al. (2021) found that urbanization can lead to SOC loss, affecting SOC sequestration. In contrast, Zhang Z. R. et al. (2022) demonstrated that when urban development reaches a higher stage, a series of ecological construction projects (e.g., increasing urban greenspaces and expanding park areas) implemented by the government can increase vegetation coverage and promote SOC accumulation. The impact of urbanization on dynamic changes in SOC is a complex and comprehensive issue, and controversial conclusions may stem from differences in research methods, scales, and geographical climatic conditions. Therefore, it is crucial to thoroughly explore and understand the dynamic changes of aggregate-related SOC in urban greenspaces in the context of urbanization. This is a key scientific issue in addressing global climate change and promoting the healthy and sustainable development of urban green ecosystems.

Soil aggregates are the basic functional units of soil structure and influence the physical and chemical properties of soil as well as biodiversity (King et al., 2019). SOC stability and aggregation are mainly determined by its spatial distribution and physical protection within soil aggregates (Tian et al., 2021). Soil aggregates contain approximately 90% SOC in the topsoil of terrestrial ecosystems and can provide physical protection, preventing direct contact between organic matter and microbial decomposition. Physical encapsulation is a primary mechanism underlying the stability of SOC (King et al., 2019). Currently, various methods are available for soil aggregate separation, enabling their classification based on chemical components such as organic matter, minerals, microorganisms, and water content (Yudina and Kuzyakov, 2019). The fractionation based on aggregate size classes is a common approach in aggregate research and is simpler and more intuitive than chemical composition. Furthermore, the size and dimensions of aggregates provide crucial information about their composition and structure, enabling a deeper understanding of their impact on ecological processes such as SOC sequestration, nutrient cycling, and gas exchange (King et al., 2019). Therefore, understanding the processes that promote soil aggregate formation and stability at the aggregate scale is crucial for enhancing SOC sequestration in urban greenspaces.

SOC stock depends on the balance between net carbon input and decomposition processes, which are influenced by various factors, including soil physicochemical properties, soil microbial community composition, climate change, land use type, and vegetation characteristics (Chen et al., 2022). Among these factors, soil microorganisms play crucial roles as carbon cycling and sequestration drivers. They are major organic matter decomposers in the soil and can directly contribute to organic matter through reproduction and mortality. In addition, they can potentially capture and store atmospheric carbon underground (Malik et al., 2018). The effects of microorganisms on SOC are closely related to the catalytic role of soil enzymes. Most soil enzymes are derived from microorganisms and play a critical intermediary role in SOC cycling, involving processes such as decomposition, transformation, and fixation (Fanin et al., 2022). Soil enzyme activity reflects the metabolic levels of soil microorganisms and indirectly indicates the processes of SOC decomposition and transformation. Overall, soil microorganisms and enzyme activity play critical roles in carbon cycling by participating in SOC decomposition and transformation processes. However, microbial-enzyme-driven SOC changes exhibit significant variability across ecosystems (Vasenev et al., 2014). Urban ecosystems have changed ecological processes and functions compared to natural ecosystems such as forests and grasslands. Intense human disturbance and resource concentrations have imposed significant pressure on the natural environment. The high spatial heterogeneity of unique biological communities and environmental climates poses challenges to SOC sequestration. Therefore, in the context of rapid urbanization, it is crucial to address how soil microorganisms and enzymes drive and affect changes in soil and aggregate-related SOC content. Understanding the effects underlying this process will provide an important theoretical basis for SOC sequestration in urban greenspaces.

Nanchang, the capital city of Jiangxi Province in China, is an important part of the Yangtze River Midstream Urban Agglomeration. Since 1995 to 2020, Nanchang has witnessed rapid urbanization and significant expansion of construction land (Zhu et al., 2023). Due to climatic influences, the main soil type in Nanchang City is red soil, which is characterized by acidity, low fertility, and stickiness. It is highly susceptible to human disturbances, leading to decreased soil porosity and inadequate organic matter content, among other adverse effects (Jin et al., 2022). Due to intense urban activities and land use changes, soil carbon storage decreased by 7.89 × 10^6^ t by calculating the land use transfer matrix of Nanchang urban agglomeration from 2000 to 2020 (Fu et al., 2023). Chen et al. (2014) uncovered that the input of large amounts of exogenous phosphorus (e.g., phosphorus from urban household waste) into the urban soils in Nanchang exacerbated the mineralization of SOC and led to a decrease in SOC stability. This phenomenon leads to substantial carbon outflows and imposes significant constraints on the sequestration function of SOC (Cai et al., 2023). Therefore, this study focused on Nanchang City as the research area to investigate the impacts of urbanization on SOC of greenspace soil and aggregates. This study aimed to explore the effects of microbial enzyme regulation of SOC transformation and sequestration. These findings provide strong scientific evidence for enhancing SOC sequestration and urban greenspace ecological services in the context of urbanization. Therefore, this study aimed to: (1) clarify the effects of urbanization on SOC content and stock of greenspace soil and aggregates; (2) explore the key effects of soil microbial enzyme driving SOC dynamics; and (3) provide basic data and management recommendations for enhancing the SOC sequestration of urban greenspace soil and aggregates from the perspective of the combined effects of microbial enzymes and other urban environmental factors.

## 2 Materials and methods

### 2.1 Overview of the study area

The study area is located in Nanchang City, the capital city of Jiangxi Province, China (28°09′∼29°11′ N, 115°27′∼116°35′ E). The average elevation of the region is 30 m, and the terrain is relatively flat. The dominant vegetation type is evergreen broad-leaved forests. It has a subtropical monsoon climate with hot and rainy summers and mild and less rainy winters. The average annual temperature is 17.5°, and the average annual precipitation is 1600–1800 mm. Nanchang has experienced rapid urbanization, with a total urban population of 6.54 million by the end of 2022 (Government of Nanchang, China).

### 2.2 Classification of urbanization intensity

The increase in impermeable surface area (ISA) caused by urbanization is an important indicator for exploring urban expansion and analyzing the quality of urban ecological environments. It has been widely applied in various fields, such as urbanization monitoring and population estimation (Huang et al., 2021). The threshold segmentation method can be used to classify ISA into different gradient zones based on construction intensity, which can then be used to analyze spatial changes in urban areas. Landsat-8, launched by NASA, is the latest satellite in the Landsat series. Compared with other satellites in the Landsat series, it can acquire data more conveniently and has a higher resolution, enabling more accurate identification of objects on the ground (Zhang et al., 2017). In this study, the spectral mixture analysis method was applied to classify Landsat-8 images, extract the spatial distribution of ISA in the study area, and divide the study area into a grid of 100 m × 100 m. The ISA proportion for each grid was calculated as an urbanization intensity indicator. The urbanization intensity within all grids was classified into three gradient levels: low (ISA < 50%), medium (50% ≤ ISA < 80%), and high (ISA ≥ 80%) (Jin et al., 2022; Wang Q. et al., 2023).

### 2.3 Soil sample collection

**FIGURE 1:**
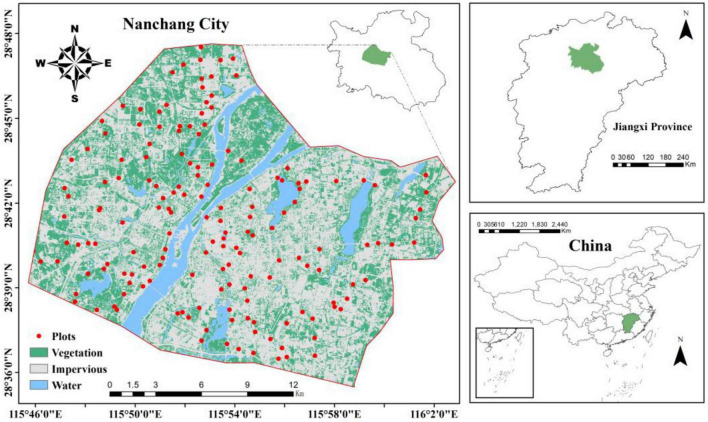
Overview of the study area.

In June 2020, 184 greenspace plots (400 m^2^, Figure 1) were established within the built-up area of Nanchang City (505 km^2^). Five sampling points were evenly distributed within each plot, and a 0–20 cm column of soil was collected using a cutting ring. Five samples from each plot were mixed, immediately weighed, and returned to the laboratory. The soil samples were divided into two parts: one part was air-dried and used to determine the SOC content and soil physicochemical properties, and the other part was stored in a freezer at −20 C for determination of soil microbial community structure and enzyme activity. Urban greenspace is artificially planned and managed, the choice of tree species is often influenced by factors such as human-made landscaping, esthetics and resistance, with a wide variety of species and spatial variation. To reduce interferences caused by various plant species, *Cinnamomum camphora* was selected for the dominant species in every urban greenspace plot.

### 2.4 Vegetation survey and soil physicochemical property determination

During the collection of soil samples, plant information within the 400 m^2^ plots was simultaneously recorded as aboveground vegetation factors, including tree species and abundance. Using these data, Shannon diversity index (SHDI) (Equation 1) and Simpson evenness index (SPEI) (Eqn 2) were calculated. Tree height was measured using a rangefinder (BOSCH GLM4000, Germany), whereas tree diameter at breast height was measured using a diameter tape at a height of 130 cm. The crown width was estimated by projecting the tree canopy. The differences in vegetation characteristics among different levels of urbanization intensity are shown in Table 1.

**TABLE 1:** Effects of urbanization on environmental factors and percentage of soil aggregates.

Factors		Urbanization		
		**Low**	**Medium**	**High**
Vegetation characteristics	TH(m)	9.52(3.94)a	8.31(2.41)a	6.81(0.41)b
	DBH(cm)	24.44(3.92)a	24.37(8.04)a	20.80(4.88)b
	CD(m^2^)	24.20(1.70)a	21.49(1.62)a	16.80(2.42)a
	SHDI	1.22(0.09)a	1.28(0.11)a	1.16(0.18)a
	SPEI	0.45(0.03)a	0.46(0.03)a	0.43(0.06)a
Soil physicochemical properties	TN(mg⋅g^–1^)	0.98(0.04)a	0.84(0.03)b	0.82(0.04)b
	TP(mg⋅g^–1^)	0.73(0.04)a	0.67(0.02)a	0.61(0.03)a
	pH	6.76(0.09)b	7.09(0.09)ab	7.29(0.16)a
	BD(g⋅cm^–3^)	1.31(0.01)b	1.35(0.01)ab	1.39(0.02)a
Percentage of soil aggregate (%)	> 2 mm	10.11(0.97)a	8.93(0.73)a	8.86(1.14)a
	1∼2 mm	10.99(0.51)a	9.88(0.51)a	11.02(0.78)a
	0.25∼1 mm	43.03(1.06)a	41.79(1.31)a	40.85(1.38)a
	0.053∼0.25 mm	16.86(0.65)b	19.46(0.83)a	20.40(1.15)a
	< 0.053 mm	19.01(1.06)a	19.94(1.22)a	18.87(1.66)a

Different lowercase letters indicate significant differences among different urbanization intensity levels (*p* < 0.05). Mean (SE). TH, Tree height; DBH, Diameter at breast height; CD, Crown width; SHDI, Shannon diversity index; SPEI, Simpson evenness index; TN, Total nitrogen; TP, Total phosphorus; BD, Bulk density. The same is below.


(1) $$\text{SHDI}=-\sum_{\text{i}=1}^{\text{S}}{\text{P}}_{\text{i}}\,\ln{\text{P}}_{\text{i}}$$

(2) SPEI=-∑Pi2

In the above formulas, P_*i*_ represents the proportion of individuals of species i to the total number of individuals in the community, and S represents the number of species.

The soil physicochemical properties were determined using the methods described by Bao (2000). The soil total nitrogen (TN) was determined using the Kjeldahl method. Soil total phosphorus (TP) was determined using the NaOH fusion-molybdenum-antimony colorimetric method. The soil pH was measured using the potentiometric method with a soil-to-water ratio of 2.5:1. Bulk density was calculated as the soil dry weight to the soil volume ratio. The differences in soil physicochemical properties among different levels of urbanization intensity are detailed by Table 1.

### 2.5 Soil aggregate fractionation and SOC determination

In this study, the soil aggregates were separated using the wet sieve method (Zhang X. R. et al., 2022). To achieve a more nuanced characterization across different aggregates size fractions, this study classified the soil aggregates into five size fractions based on previous researches (Arai et al., 2018; Lin et al., 2023): > 2 mm, 1–2 mm, 0.25–1 mm, 0.053–0.25 mm, and < 0.053 mm (Table 1). The SOC content of bulk soil and each soil aggregate was measured using the dichromate titration method (Bao, 2000), and the SOC stock and its contribution rate were calculated for each aggregate using the following formulas:

The formula for calculating SOC-stock (g⋅m^–2^) is as follows (Shao et al., 2005):


(3) SOC-stock=(D×BD×SOC)/10

The formula for calculating the Stock of SOCn (g⋅m^–2^) in each soil aggregate size fraction is as follows (Zhong et al., 2020):


(4) $$\mathrm{Stock}\,\mathrm{of}\,{\mathrm{SOC}}_{\text{n}}=\left(\text{D}\times \text{BD}\times {\text{W}}_{\text{n}}\times {\text{SOC}}_{\text{n}}\right)/10$$

The SOC contribution rate, which represents the contribution of SOC in different sized aggregates to the total SOC content. W is given by the following formula (Fan et al., 2021):


(5) $$\text{W}=\left({\text{SOC}}_{\text{n}}\times {\text{M}}_{\text{n}}\right)/\left(\sum_{\text{n}\times 1}^{\text{n}}{\text{SOC}}_{\text{n}}\times {\text{M}}_{\text{n}}\right)\times 100\%$$

In the above formulas: SOC represents the SOC content (g⋅kg^–1^); D represents the soil depth (cm); BD represents the soil bulk density (g⋅cm^–3^); W_*n*_ represents the percentage content (%) of soil aggregates of n size; M_*n*_ represents the mass (g) of soil aggregates of n size; SOC_*n*_ represents the SOC content (g⋅kg^–1^) in the soil aggregates of n size.

### 2.6 Determination of soil microbial community structure

The microbial community structure was determined using the phospholipid fatty acid (PLFA) method. PLFAs were extracted and analyzed per the method described by Zelles et al. (1992). Each PLFA concentration was internally standardized with a methyl ester of 19:0, and standard nomenclature was used. Based on existing research results, this study summarizes the microorganisms represented by several common PLFAs in the soil. Fungi are represented by: 18:1ω9c, 18:2ω6c, 18:3ω6c (Dickens et al., 2013); arbuscular mycorrhizal fungi (AMF) are represented by: 16:1ω5 (Johansen et al., 1996); actinomycetota (Act) are represented by: 10Me17:0, 10Me16:0, 10Me18:0 (Li et al., 2020); gram-positive bacteria (G+) are represented by: i14:0, i15:0, a15:0, a17:0, i16:0, i17:0 (Li et al., 2015); gram-negative bacteria (G-) are represented by: 18:1ω7c, 16:1ω7c, cy17:0, cy19:0 (Li et al., 2015); protozoa (Pro) are represented by: 20:3ω6, 20:4ω6 (Wan et al., 2019).

### 2.7 Determination of soil enzymatic activities

In this study, five enzyme activities were measured for analyzing the SOC dynamics. Urease (URE) activity was determined using the sodium hypochlorite-phenol red colorimetric method (Guan, 1986), catalase (CAT) activity was determined using the potassium permanganate titration method (Guan, 1986), and β-1,4-glucosidase (BG), N-acetylglucosaminidase (NAG), and acid phosphatase (AP) activities were determined using the microplate fluorescent substrate labeling method (Saiya-Cork et al., 2002).

### 2.8 Data analysis

In this study, the Kolmogorov-Smirnov test was used to analyze whether all data followed a normal distribution, and a logarithmic conversion was conducted for data that did not meet the normal distribution. One-way ANOVA and Duncan’s method were used to reveal the significance of differences among various factors related to different urbanization levels (*p* < 0.05). Redundancy analysis (RDA) was conducted to explore the relationships between soil microbial community structure, enzyme activities, and aggregate-related SOC content. In addition, structural equation model (SEM) was employed to further explore the complex coupling relationships between urbanization intensity, vegetation factors, soil physicochemical properties, soil microbial community structure, enzyme activities, and aggregate-related SOC. Before the analysis, principal component analysis was conducted on the data of vegetation characteristics, soil physicochemical properties, soil microbial community structure, and enzyme activities to reduce the dimensionality of each dataset and obtain the main information and eigenvalues of each group of data. This approach aims to improve the model fit while retaining the primary features of the original data. The model was constructed using the SPSS Amos Graphics software (IBM Corp., Armonk, NY, USA). Data analysis and graphical representations were performed using Origin 2018, SPSS 26.0, ArcGIS 10.0, and Canoco 5.0.

## 3 Results

### 3.1 Impact of different urbanization intensities on aggregate-related SOC content and stock

**FIGURE 2:**
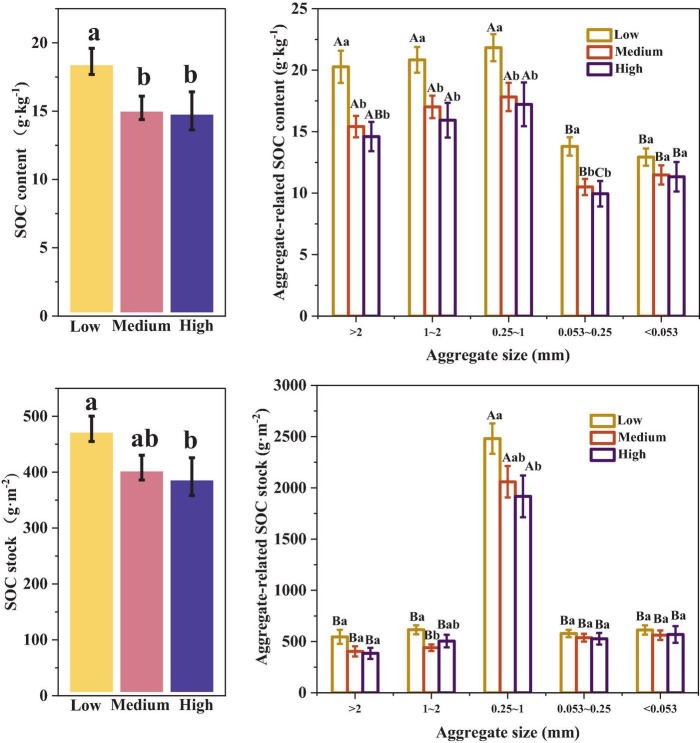
Differences in aggregate-related SOC content and stock under different urbanization intensities. Different capital letters indicate significant differences within the same urbanization intensity level (*p* < 0.05), whereas different lowercase letters indicate significant differences among different urbanization intensity levels (*p* < 0.05).

As shown in Figure 2, there were significant differences in SOC content among the different urbanized areas (*p* < 0.05). The SOC content in low urbanization areas was significantly higher than that in medium and high urbanization areas, ranging from 3.40–3.62 g⋅kg^–1^ (*p* < 0.05). The pattern was similar for the aggregate-related SOC content. In low urbanization areas, the SOC content of > 0.053 mm aggregates was significantly higher than that in medium and high urbanization areas by 26–39% (*p* < 0.05). However, there was no significant difference in SOC content of < 0.053 mm aggregates across different urbanization intensity levels (*p* > 0.05). In medium and low urbanization areas, the SOC content of > 0.25 mm aggregates was significantly higher than that of < 0.25 mm aggregates (*p* < 0.05). In high urbanization areas, the highest SOC content was observed in aggregates with sizes in the range of 1–2 mm and 0.25–1 mm, with values of 15.93 g⋅kg^–1^ and 17.22 g⋅kg^–1^, respectively. These values were significantly higher than the SOC content of < 0.053 mm aggregates. Additionally, the SOC content of < 0.053 mm aggregates was significantly higher than that of aggregates ranging from 0.053 to 0.25 mm (*p* < 0.05).

At the same time, SOC stock was significantly affected by urbanization. The SOC stock in high urbanization areas was reduced by 85.48 g⋅m^–2^ compared to low urbanization areas. In the 0.25–1 mm and 1–2 mm aggregates, the SOC stocks were significantly higher in the low urbanized area compared to both the high urbanized area and the medium urbanized area, respectively (*p* < 0.05). However, urbanization did not have a significant effect on SOC stock for other aggregates (*p* > 0.05). Additionally, the SOC stock of 0.25–1 mm aggregates was the highest (1917.13 g⋅m^–2^–2480.96 g⋅m^–2^), significantly higher than the other aggregates, making it a key contributor to SOC stock (*p* < 0.05).

**FIGURE 3:**
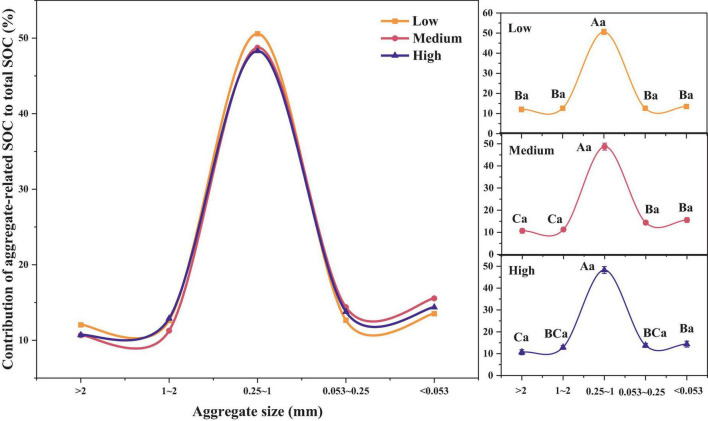
Differences in SOC contribution rates among different urbanization intensity levels. Different capital letters indicate significant differences within the same urbanization intensity level (*p* < 0.05), whereas different lowercase letters indicate significant differences among different urbanization intensity levels (*p* < 0.05).

As shown in Figure 3, the contribution of the SOC content in different soil aggregates to the total SOC content was relatively similar and did not show significant differences across different urbanization intensities (*p* > 0.05). As for different aggregate sizes, we can once again confirm that in regions with different urbanization intensity levels, the contribution rate of SOC of 0.25–1 mm aggregates is the highest (48.29–50.58%), significantly higher than other aggregates (*p* < 0.05). In addition, in medium urbanization areas, the SOC contribution rate of < 0.25 mm aggregates was significantly higher than that of > 1 mm aggregates. In highly urbanized areas, the SOC contribution rate of < 0.053 mm aggregates was significantly higher than that of > 2 mm aggregates (*p* < 0.05).

### 3.2 The effects of different urbanization intensities on soil microbial community structure and soil enzyme activities

**FIGURE 4:**
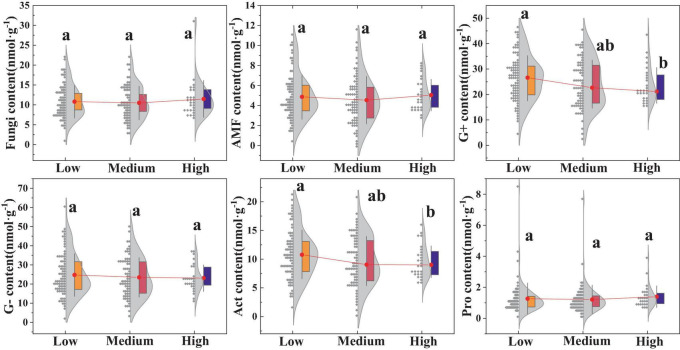
Impact of urbanization on soil microorganisms. Different lowercase letters indicate significant differences among different urbanization intensity levels (*p* < 0.05). AMF, Arbuscular mycorrhizal fungi; G–, Gram-negative bacteria; G+, Gram-positive bacteria; Act, Actinomycetota; Pro, Protozoa. The same is below.

There were certain differences in the soil microbial community structures among the different urban regions (Figure 4). The results indicated that the biomass of G+ and Act in high urbanized areas was significantly lower compared to low urbanized areas (*p* < 0.05), whereas the biomass of G-, fungi, AMF, and Pro showed no significant differences (*p* > 0.05).

**FIGURE 5:**
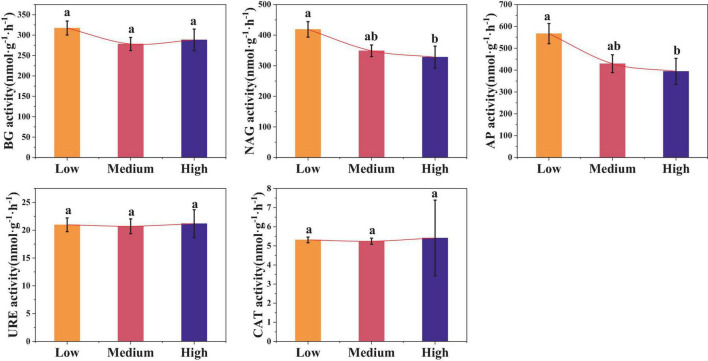
Differences in soil enzyme activities among different levels of urbanization intensity. Different lowercase letters indicate significant differences among different urbanization intensity levels (*p* < 0.05). BG, β-1,4-glucosidase; NAG, N-acetylglucosamine hydrolase; AP, Acid phosphatase; URE, Urease; CAT, Catalase; The same is below.

Urbanization also affected the soil enzyme activity (Figure 5). Compared to low urbanized areas, the activities of NAG and AP in high urbanized areas were significantly reduced by 21.53 and 33.33%, respectively (*p* < 0.05). However, there were no significant differences in the BG, URE, and CAT activities among the three urban areas (*p* > 0.05). The average values of BG, URE, and CAT in urban areas are 294.66 nmol⋅g^–1^⋅h^–1^, 20.90 nmol⋅g^–1^⋅h^–1^, and 5.30 nmol⋅g^–1^⋅h^–1^, respectively.

### 3.3 Correlation analysis between soil microbial community structure, soil enzyme activities, and SOC content in different soil aggregates

**FIGURE 6:**
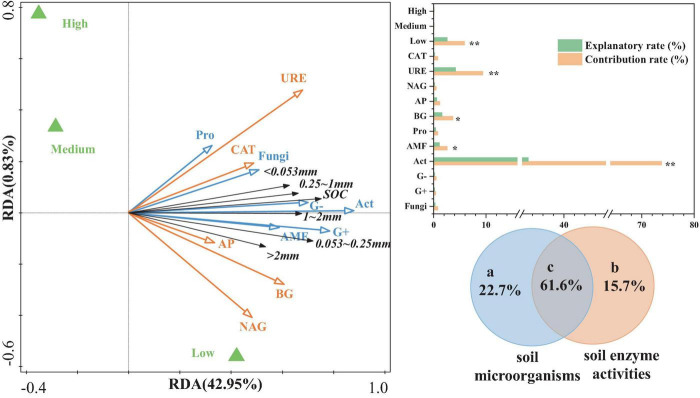
RDA and VPA of aggregate-related SOC in relation to soil microorganisms and enzymes. ** represents *p* < 0.01, * represents *p* < 0.05.

Canonical RDA was used to analyze the relationship between aggregate-related SOC content as the response variable and various soil enzyme factors and microbial community structure as explanatory variables (Figure 6). The results indicated that the first axis explained 42.95% of the variation in aggregate-related SOC content, while the second axis explained extremely low. Together, these two axes explained a cumulative total of 43.78% of the variation. Most of the soil microbial community compositions and soil enzyme activities were positively correlated with aggregate-related SOC content. Compared to soil enzyme activities, the composition of soil microbial communities contributed more significantly to aggregate-related SOC content, particularly Act, G+, G−, and AMF. Among these, Act (33.2%), URE (4.2%), BG (2.6%), and AMF (1.1%) showed significant explanatory power (*p* < 0.05). Additionally, the effects of NAG, AP, and BG were more pronounced in areas with low urbanization.

To further validate and elucidate the comprehensive explanatory capacity of soil microbial community structure and enzyme activities (two sets of environmental parameters) on the variation in aggregate-related SOC content, this study conducted variation partitioning analysis (VPA) (Figure 6). The interaction between soil microbial community structure and soil enzyme activities demonstrated the highest explanatory power (61.6%) for variations in the aggregate-related SOC content. The soil microbial community structure (22.7%) was followed by soil enzyme activity (15.7%). Therefore, the mutual interaction between soil microbes and enzymes plays a crucial role in the aggregate-related SOC content.

### 3.4 Structural equation modeling analysis

**FIGURE 7:**
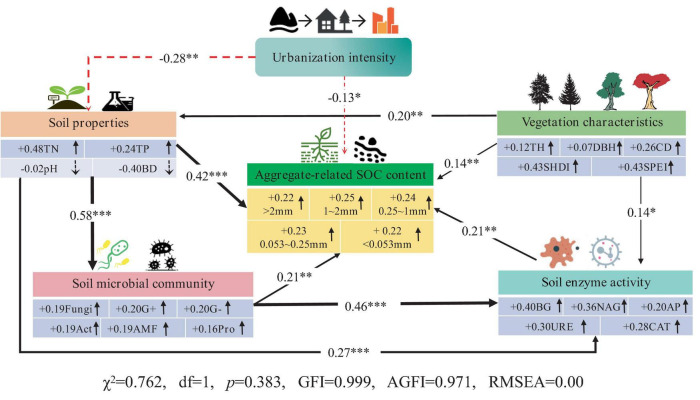
Structural equation model analysis result. The red dotted line indicates a negative influence, and the solid black line indicates a positive influence; *** represents *p* < 0.001, ** represents *p* < 0.01, and * represents *p* < 0.05.

The use of SEM to elucidate the interactive effects among urbanization intensity, soil microbial community structure, soil enzyme activities, environmental factors, and aggregate-related SOC content is important. As shown in Figure 7, urbanization directly led to a decrease in the aggregate-related SOC content (−0.13). Urbanization can also indirectly affect aggregate-related SOC through other factors, including the following pathways: (1) Urbanization can indirectly reduce aggregate-related SOC content by altering soil physicochemical properties (reducing TN, TP, increasing bulk density), with a pathway coefficient of −0.12 (−0.28 × 0.42). (2) Urbanization can indirectly decrease soil microbial biomass and enzyme activity by altering soil physicochemical properties, thereby reducing the aggregate-related SOC content. The pathway coefficients are −0.03 (−0.28 × 0.58 × 0.21) and −0.02 (−0.28 × 0.27 × 0.21), respectively. (3) Urbanization can indirectly decrease the aggregate-related SOC content by altering soil physicochemical properties, which in turn affects soil microbial biomass and subsequently reduces soil enzyme activities. The pathway coefficient is −0.02 (−0.28 × 0.58 × 0.46 × 0.21). Vegetation characteristics had the opposite effect on aggregate-related SOC compared to urbanization. Increasing tree height, canopy width, SHDI and SPEI can directly enhance the aggregate-related SOC content. Additionally, they can indirectly increase aggregate-related SOC content (0.14 × 0.21 = 0.03) by promoting soil enzyme activities (BG, NAG, AP, URE, and CAT). Therefore, the decrease in aggregate-related SOC content due to urbanization is a combination of direct and indirect effects primarily driven by soil microbes and enzymes.

## 4 Discussion

### 4.1 Urbanization can directly or indirectly decrease aggregate-related SOC by altering soil physicochemical properties

This study found a significant decrease in SOC content of bulk soil and > 0.053 mm aggregates with increasing urbanization intensity. A comprehensive analysis revealed that the decrease in SOC caused by urbanization can be attributed to both direct and indirect effects. Similar to this study, Luo et al. (2021) demonstrated that urbanization leads to SOC loss. During urbanization, land for construction purposes often undergoes large-scale land leveling and filling, which can disrupt the original soil structure and composition of soil aggregates (Cai et al., 2023). At the same time, artificial surfaces such as buildings and roads in urban areas often lack organic matter accumulation, which hinders SOC sources. The increase in impermeable surfaces leads to hotspot formation in urban areas where SOC content is lost as the sealed soil becomes unable to retain carbon. Additionally, factors, such as reduced litter input and increased urban temperatures, can accelerate the carbon decomposition rate, ultimately resulting in SOC content decrease (Cai et al., 2023). Therefore, land-use change significantly alters the distribution of different soil aggregates, thereby affecting aggregate stability and the association between aggregates and SOC (Yu et al., 2023).

Furthermore, the results indicate that SOC of > 0.25 mm coarse aggregates is significantly higher than in small aggregates in urban areas, suggesting that the carbon physically protected by larger aggregates plays a crucial role in SOC accumulation. According to the aggregate hierarchy concept proposed by Tisdall and Oades (1982), soil aggregates can form larger aggregates by binding mineral particles and microaggregates through multi-stage binding of substances such as polysaccharides and fungal hyphae. During this process, larger aggregates tended to encapsulate more SOC than the microaggregates. Therefore, it is widely recognized in the scientific community that soil macroaggregates contain more SOC, which is consistent with our findings.

Significant differences in SOC stocks were found only between 0.25–1 mm and 1–2 mm aggregates among different urbanization intensities, which were closely related to the SOC content in each aggregate size fraction. As the 0.25–1 mm aggregate fraction had the largest mass proportion (Table 1), it contributed the highest SOC stock and proportion, serving as the primary SOC accumulation form, particularly in low urbanization regions. It is worth mentioning that the variations in the contribution of aggregate-related SOC between different urbanization regions were higher in moderate and high urbanization areas than in low urbanization areas. Tian et al. (2020) found that rapidly developing urban areas exhibited high spatial heterogeneity in SOC density. This indicates that urban ecosystem complexity leads to greater spatial variation in the contribution of SOC to macroaggregates in highly urbanized regions, making it even more complex and anomalous.

The decrease in SOC caused by urbanization is closely related to the indirect effects of soil physicochemical properties. Research has shown that an increase in impermeable surfaces hinders or weakens the exchange of matter and energy between the soil and atmosphere, hydrosphere, and biosphere, leading to changes in the physical and chemical properties of the soil (Pouyat et al., 2007). This study found that an increase in urbanization intensity led to an increase in soil bulk density and a decrease in TN and TP content, indirectly resulting in a reduction in the SOC content of soil aggregates. Soil physicochemical properties play crucial roles in SOC formation and transformation, and a decline in soil quality inevitably has adverse effects on SOC sequestration. Nitrogen and phosphorus are the two major nutrients required for plant growth, and they play crucial regulatory roles in biological activity and organic matter decomposition processes in the soil. Numerous studies have shown that increasing the nitrogen and phosphorus supply can promote SOC sequestration. Luo et al. (2023) conducted an integrated analysis of 536 relevant studies on terrestrial ecosystems to explore the relationship between phosphorus supply and SOC content. They found that increasing the phosphorus supply enhanced SOC content and increased soil nitrogen availability. However, soil bulk density generally has a negative effect on SOC content. High bulk density affects soil permeability and water movement, thereby reducing the formation and stability of soil aggregates and decreasing their capacity for SOC content (Reijneveld et al., 2023). Overall, with an increase in urbanization intensity, sealed surface expansion alters the supply-demand cycle of soil organic matter and changes soil physicochemical properties, thereby affecting SOC cycling in urban ecosystems.

### 4.2 Urbanization influences the dynamic changes of aggregate-related SOC through the alteration of soil microbial community structure and enzyme activity

Based on previous studies, this study established the connections between environmental factors, soil microbial community structure, enzyme activity, and aggregate-related SOC. The results confirmed that the soil microbial community structure and enzyme activity played regulatory roles in SOC cycling and stock in urban ecosystems. RDA revealed that soil microbes (Fungi, G+, G−, Act, AMF, Pro) and enzymes (BG, NAG, AP, URE, and CAT) were positively correlated with aggregate-related SOC, which is consistent with previous research findings. Mason et al. (2023) suggested that soil microorganisms play a direct role in SOC sequestration by assimilating plant-derived carbon, contributing to microbial biomass, and producing recalcitrant organic matter. They also indirectly influence SOC sequestration by promoting soil enzyme secretion. VPA reveals the critical importance of the mutual interactions between soil microbes and enzymes in relation to aggregate-related SOC content, with a higher contribution from soil microbes compared to enzymes.

Microorganisms provide substrates and energy sources for organic matter and play a significant role in organic matter decomposition and transformation through diverse metabolic and biotransformation processes (Malik et al., 2020). Soil enzymes, on the other hand, primarily catalyze and regulate these processes based on microbial activity. Microorganisms and enzymes jointly drive the carbon input into soil aggregates. SEM analysis further confirmed the significant contribution of soil microbial community structure and enzyme activity to aggregate-related SOC, whereas urbanization inhibited the positive effects of microorganisms and soil enzymes.

With the increasing urbanization intensity and greater human disturbance, there was no significant change in fungal and AMF biomass, whereas bacterial biomass significantly decreased. This indicates that soil bacterial communities are more susceptible to environmental factors than fungal communities (Xiao et al., 2018). This difference in community structure may be attributed to specific growth strategies. Compared with fungi, bacteria are more adaptable to changes in nutrient utilization efficiency, have shorter turnover times, and are more sensitive to variations in soil moisture and nutrient availability (Keiblinger et al., 2010). G+, an important soil bacterial community component, was significantly higher in low urbanization areas than in other regions. Although G+ can adapt to diverse environmental systems, it tends to slowly decrease in environments with poor soil quality, characterized by low nitrogen and phosphorus levels and high bulk density. Conversely, in resource-rich environments, they can exhibit rapid growth and reproduction (Fanin et al., 2019). According to the Act, they typically thrive on recalcitrant polymers, such as lignocellulose. In highly urbanized areas, a decrease in vegetation coverage and litter removal can lead to a decline in Act biomass (Kong et al., 2011). Numerous studies have indicated that Act exhibits minimal growth under compact soil conditions (Naylor et al., 2020). However, as a widely distributed bacterium in soil, Act has a relatively high biomass. Carbon-rich biomass in the cellular bodies can provide a persistent carbon source for aggregate-related SOC accumulation (Jiao et al., 2021). Both G+ and Act promote SOC formation and stock by secreting extracellular polysaccharides. These polysaccharides bind to soil particles and organic matter, forming cohesive substances that facilitate soil aggregate formation and SOC sequestration and stabilization (Wu et al., 2023). Reischke et al. (2014) indicated in their research that SOC formation depends on the involvement of G+, G−, and Act. To some extent, their biomass reflects SOC content, and they, along with fungi, AMF, and Pro, form complex microbial communities. Through interactions and synergistic metabolism, they promote the formation of soil aggregates and the stability of SOC. Therefore, urbanization has a negative impact on microorganisms, especially bacteria, and serves as a critical factor in limiting the functionality of SOC sequestration.

Soil enzymes indirectly reflect soil microbial activity and serve as the primary metabolic driving force for soil biochemical processes (Yang et al., 2020). Different land-use patterns and forest vegetation characteristics in areas with varying degrees of urbanization can lead to changes in plant litter accumulation, soil physicochemical properties, root exudates, and other factors. These changes can cause spatial variation in microbial communities, resulting in alterations in soil enzyme activity (Lucas-Borja et al., 2016). This study found a significant decline in NAG and AP activities with increasing urbanization intensity. NAG is an enzyme involved in nitrogen acquisition, and there is a significant positive correlation between soil TN content and NAG activity. TN loss from urban greenspace soils (Table 1) inhibits NAG activity (Chen et al., 2017). Additionally, AP activity was found to be highest in low-urban areas. This could be attributed to the richer vegetation distribution in these areas, where plants have a higher phosphorus demand, resulting in stronger AP activity. The substantial organic matter produced by plant litter also stimulates AP secretion (Qiao et al., 2019). Soil enzymes are active participants in soil biochemical processes and catalyze organic matter decomposition (Yang et al., 2020). The reduction in enzyme activity inevitably led to a decrease in the aggregate-related SOC content. NAG, AP, and BG are involved in the decomposition of soil organic matter. NAG primarily catalyzes the degradation of various organic compounds, including polysaccharides, proteins, and chitin. AP hydrolyses organic phosphates and converts them into inorganic phosphates. BG degrades carbohydrates, especially lignocellulose, and releases organic acids, CO_2_, and other metabolites (Yang et al., 2020). Substances such as SOC and organic phosphates can be released through the action of these enzymes. Additionally, soil enzymes produce extracellular adhesive substances that can envelop these materials, promoting aggregate formation and stability, and enhancing SOC sequestration (Zhao et al., 2023). In addition, soil enzymes participate in the transformation of organic matter. Enzymes, such as URE and CAT, catalyze chemical reactions between organic substances, converting SOC into other forms of carbon compounds. This transformation enhances the stability of SOC, reduces its degradation rate in the soil, and promotes its sequestration (Zhao et al., 2023). In summary, during urbanization, a decline in soil quality has a negative impact on both microorganisms and soil enzymes, ultimately weakening the SOC sequestration capacity of urban greenspace soils.

### 4.3 Insights

Climate change poses a significant environmental challenge for humanity. As a major emitter of CO_2_, China solemnly announced at the 75^th^ United Nations General Assembly its commitment to peaking CO_2_ emissions before 2030 and striving to achieve carbon neutrality by 2060. Cities, as important terrestrial ecosystem components, are the primary spatial areas where carbon emissions occur and are also the main entities for carbon accounting and policymaking toward carbon neutrality (Wang Y. N. et al., 2023). Previous research on carbon sequestration has predominantly focused on forests, grasslands, wetlands, and oceans (Yang et al., 2022), often regarding cities as major carbon sources, and overlooking their role as carbon sequestration in urban ecosystems. Vegetation, soil, and water systems in urban areas possess carbon absorption capabilities and are crucial urban carbon sequestration components (Zhuang et al., 2023). As the largest carbon reservoir in terrestrial ecosystems, the soil stores four times more SOC than vegetation. Even minor SOC losses can accelerate climate change (Zhang et al., 2021). However, with urban expansion, vegetation cover decreases, and the soil structure is disrupted, compacted, and contaminated, leading to intensified nutrient loss. These factors severely limit the function of urban soil as a carbon sequestration. Therefore, the promotion of carbon sequestration in urban greenspace soils is an urgent issue. Traditional research has mainly focused on the input of plants and organic matter decomposition dividing SOC stock into processes, such as carbon allocation from photosynthesis and soil respiration. However, in recent years, increasing evidence suggests that process models built based on traditional approaches do not accurately reflect the total SOC (Tao et al., 2023). Microorganisms may seem insignificant at the macroscopic scale but are crucial factors that cannot be ignored in the carbon cycling process.

This study reveals the driving factors of SOC sequestration in urban greenspaces from the perspective of microbial-enzyme interactions, providing a theoretical basis for enhancing the carbon sequestration function in cities. Soil microorganisms and enzyme activities can effectively promote SOC sequestration. Soil nutrients (TN and TP) are important limiting factors, and appropriate fertilization during urban development can promote plant growth, enhance soil nutrients, create suitable environmental conditions for microbial survival and reproduction, and increase soil enzyme activity. The common high bulk density during urbanization reduces microbial habitat space and mobility, which is unfavorable for SOC sequestration. In urban greenspaces, the dispersal of human traffic, timely loosening of soil, and the use of soil amendments (such as organic matter and humic acid) can increase microbial biomass and enhance hydrolytic enzyme secretion thereby promoting SOC accumulation. The effect of microorganisms on carbon sequestration also depends on plant participation, resulting in a close interaction between the two. As crucial soil carbon cycling components, larger tree heights and canopy sizes provide more abundant litter (such as leaves and branches). Increased SHDI and SPEI values indicate enhanced plant diversity, which can increase the variety and quantity of plant residues in the soil. Plant residues and litter are rich in SOC and release organic acids and other organic substances during the degradation process (Chen et al., 2023). Plant residues and litter also improve the physical and chemical properties of the soil, promote soil nutrient cycling, provide abundant carbon sources and nutrients for soil microorganisms, and stimulate decomposition enzyme production and activity, thereby promoting SOC input (Qian et al., 2023). Furthermore, the growth and extension of plant roots can alter the physical structure of the soil, stabilizing aggregate formation and introducing SOC into the soil through root exudates and residual root tissues (Panchal et al., 2022). Therefore, emphasizing the close interaction between microorganisms, soil enzymes, soil properties, and upper vegetation is key to mitigating SOC loss and maintaining the SOC sequestration function.

## 5 Conclusion

This study revealed the effects by which soil microbiota and enzymes, as driving factors, influence aggregate-related SOC content under different urbanization intensity levels in Nanchang, China. As urbanization intensity increased, both the content and stock of SOC demonstrated a significant decline, and the SOC content as well as stock of various soil aggregates also decreased, with the 0.25–1 mm aggregates, which had the highest mass proportion, exhibiting the greatest response to urbanization. Concurrently, the soil microbial community and enzyme activities were also influenced by urbanization, with reductions observed in the biomass of G+ and Act, as well as the activities of NAG and AP. There was a significant positive correlation among soil microbiota, enzymes, and aggregate-related SOC, with Act, URE, BG, and AMF showing the strongest correlations. Compared to enzymes, soil microbiota makes a greater contribution to aggregate-related SOC content; however, the interactive effects between soil microbiota and enzymes play a crucial role in the aggregate-related SOC content. SEM analysis further revealed that urbanization not only directly reduces aggregate-related SOC but also indirectly affects soil microbiota and enzyme activities by reducing TN and TP and increasing bulk density, thereby leading to a decrease in aggregate-related SOC content. Conversely, vegetation characteristics, such as tree height, canopy width, and diversity indices, can mitigate the negative effects of urbanization and directly or indirectly promote carbon sequestration in soil aggregates. Therefore, future efforts should focus on strengthening urban forest management, preserving existing large trees, enriching vegetation communities to provide suitable habitat conditions for soil microbiota, and fully harnessing the positive role of soil microbiota and enzymes in the carbon sequestration of urban soils.

## Data availability statement

The original contributions presented in the study are included in the article/supplementary material, further inquiries can be directed to the corresponding author.

## Author contributions

FZ: Data curation, Formal Analysis, Software, Visualization, Writing−original draft, Writing−review and editing. JZ: Investigation, Writing−original draft, Data curation. YZ: Methodology, Resources, Writing−review and editing. CC: Investigation, Methodology, Writing−original draft. WL: Methodology, Project administration, Writing−review and editing. QW: Conceptualization, Funding acquisition, Investigation, Resources, Writing−original draft, Writing−review and editing. WW: Funding acquisition, Resources, Writing−review and editing. HW: Funding acquisition, Project administration, Writing−review and editing. XJ: Funding acquisition, Project administration, Writing−review and editing. RY: Investigation, Software, Writing−review and editing.
